# Petrology and geochemistry dataset of lava from the Ijen Crater and Mount Blau, Banyuwangi, East Java, Indonesia

**DOI:** 10.1016/j.dib.2019.104765

**Published:** 2019-11-06

**Authors:** Aditya Pratama, Handayani Hadiyat, Fadhli Ramadhana Atarita, Satria Bijaksana, Djoko Santoso, Mirzam Abdurrachman, Silvia Jannatul Fajar

**Affiliations:** a Faculty of Mining and Petroleum Engineering, Institut Teknologi Bandung, Bandung 40132, Indonesia; b Faculty of Earth Sciences and Technology, Institut Teknologi Bandung, Bandung 40132, Indonesia

**Keywords:** Lava, Ijen crater, Mount blau, Whole rock geochemistry, Rock texture, Indonesia

## Abstract

This article presents rock texture and mineralogy, as well as major and trace elements of lava from Ijen Crater and Mount Blau, Ijen Volcanic Complex (IVC), East Java, Indonesia related to article entitled “Rock Magnetic, Petrography, and Geochemistry Studies of Lava at the Ijen Volcanic Complex (IVC), Banyuwangi, East Java, Indonesia” [1]. Six lava samples were taken from three lava flows that are the product of the eruption of the Ijen Crater and three lava samples from a lava flow that is the product of the eruption of Mount Blau. The samples were crushed and used for measuring major and trace elements using XRF method. Meanwhile, the thin sections of all samples were used to analyze rock texture and mineralogy. These data are invaluable in identifying the lithology, tectonic setting, and magmatism process through the analysis of total silica alkaline and Harker diagram. Other researchers can analyze the other diagrams and graphs to know in more detailed information as intended. On the other hand, the data can be used as a comparison for other lava products from different eruption sources.

**Table:** Specifications Table

Subject	Geochemistry and Petrology
Specific subject area	Whole rock geochemistry, mineralogy, and rock texture of lava
Type of data	Table Image Graph Figure
How data were acquired	1. Jaw Crusher made by Retsch, Germany and Pulveriser made by Fritsch, Germany were used for crushing the rock be powder. 2. Carbolite muffle furnace made by Carbolite Gero, UK was used for heating and weighing the samples, respectively. 3. Thermo Scientific ARL 990 (X-Ray fluorescence instrument) made by Thermo Electron Corporation, Switzerland was used to measure whole rock geochemistry (major and trace elements). 4. Microsoft Excel was used to make the graphs. 5. Ci-POL polarizing microscope made by Nikon, Tokyo, Japan was used to analyze the mineralogy and rock texture. 6. OptiLab microscope camera with optiLab viewer and image raster 3 software made by PT Miconos, Jogjakarta, Indonesia were used to take and analyze the thin section images.
Data format	Raw Analyzed
Parameters for data collection	The samples which were used for the whole rock geochemistry measurement should be crushed to a size of ≤50 μm. All of the grains should have the relatively same size. This measurement was conducted at room temperature. For the petrography analyses, all of the samples were prepared as thin sections with thickness of about 0.03 mm.
Description of data collection	Lost on ignition (Lol) measurements were done with heating the samples to the temperature of 1000 °C. Samples masses before and after heating were measured to know the weight loss. Major and trace elements measurements were done using X-Ray Fluorescence (XRF) method. Measured major elements are SiO_2_, TiO_2_, Al_2_O_3_, Fe_2_O_3_, MnO, MgO, CaO, Na_2_O, K_2_O, and P_2_O_5_. Measured trace elements are Ba, Cu, Zn, Rb, Sr, Co, Y, and V. Meanwhile, Mineralogy and rock texture analysis were done with the observation of the samples' thin section through a polarizing microscope.
Data source location	Institution: (1) Center for Geological Survey (CGS) of Indonesia, (2) Faculty of Earth Sciences and Technology, Institut Teknologi Bandung City: Bandung Province: West Java Country: Indonesia Sampling locations: Ijen Crater and Mount Blau, Ijen Volcanic Complex City: Banyuwangi Province: East Java Country: Indonesia
Data accessibility	The data are available within this article.
Related research article	Author's name: Aditya Pratama, Satria Bijaksana, Mirzam Abdurrachman, and Nono Agus Santoso Title: Rock Magnetic, Petrography, and Geochemistry Studies of Lava at the Ijen Volcanic Complex (IVC), Banyuwangi, East Java, Indonesia Journal: Geosciences 2018, 8, 183 https://doi.org/10.3390/geosciences8050183

**Table:** 

**Value of the Data** • The data presented in this article can be used in the study of petrology and geochemistry of the lava from the Ijen Crater and Mount Blau, mainly in lithology, tectonic setting, and magmatism process. • Researchers that are currently working in the field of volcanology, mainly in geochemistry, mineralogy, and rock texture, can use this data according to their studies. • The data presented in this article can be compared with other data to understand the similarities and differences with other lava flow from different eruption sources. With further statistical analyses, more detailed information about lithology, tectonic setting, and magmatism can be obtained. • The data presented in this article complement the data obtained from the previous study [1].

## 1 Data

**Fig. 1:**
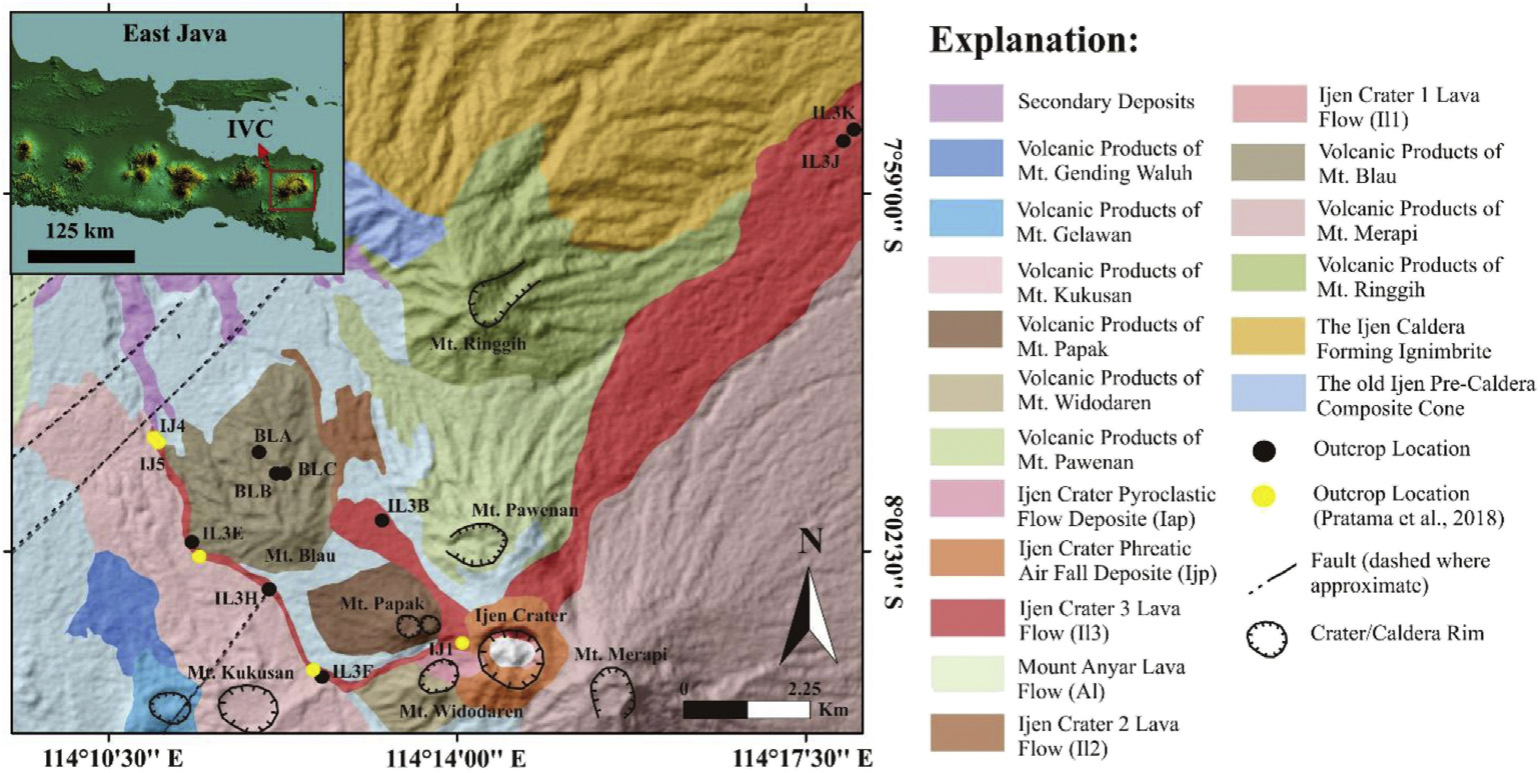
The distribution of the volcanic products from different eruption centers [2].

Fig. 1 shows the geological map from the study area. Sampling locations are shown on the map as the black dots. Samples were taken from four different lava flows. Three lava flows are the product of the eruption of the Ijen Crater (IL3B, IL3E, IL3F, IL3H, IL3J, and IL3K), meanwhile, one other lava flow is the product of Mount Blau's eruption (BLA, BLB, and BLC). The coordinates of the outcrops can be seen in Table 1.

**Table 1:** Geographic locations of sampling points.

Sample ID	Latitude	Longitude
IL3B	8° 02′ 05.807″ S	114° 13′ 11.900″ E
IL3E	8° 02′ 09.018″ S	114° 11′ 11.070″ E
IL3F	8° 03′ 43.216″ S	114° 12′ 41.929″ E
IL3H	8° 03′ 00.601″ S	114° 12′ 02.896″ E
IL3J	7° 59′ 09.884″ S	114° 17′ 29.050″ E
IL3K	7° 59′ 08.892″ S	114° 17′ 31.504″ E
BLA	8° 00′ 49.830″ S	114° 11′ 55.993″ E
BLB	8° 00′ 56.335″ S	114° 11′ 55.949″ E
BLC	8° 00′ 44.118″ S	114° 12′ 21.778″ E

**Fig. 2:**
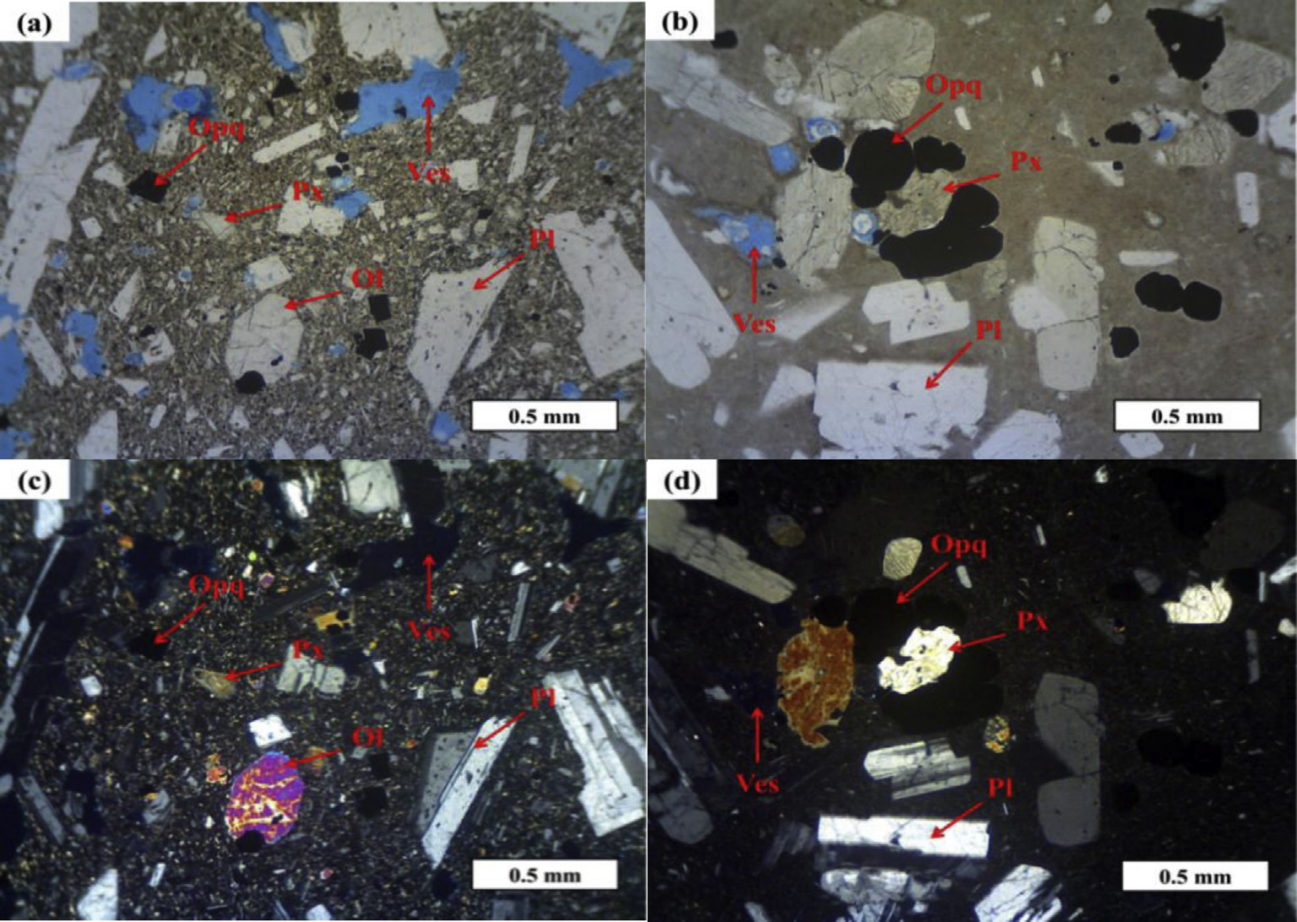
Thin sections show the differences in texture in parallel nicol mode of (**a**) IL3B; (**b**) BLA and cross nicol mode of (**c**) IL3B; (**d**) BLA. Mineral abbreviations: Pl = plagioclase; Px = pyroxene, Ves = vesicular, Ol = olivine.

The thin sections of the samples from the Ijen Crater and Mount Blau are shown in Fig. 2. All samples from the Ijen Crater have porphyritic texture with phenocryst 25–35%, as plagioclase (15–21%); pyroxene (3–9%); olivine (1%); and opaque minerals (2–7%) with the size of 0.1–5.25 mm with groundmass that is consisted of minerals such as plagioclase microlite (15–37%); pyroxene (7–15%); olivine (1%); opaque minerals (2–4%); and glass (12–39%). Meanwhile, the lava sample from Mount Blau has phenocryst approximately 35–40%, consisted of plagioclase (22–30%); pyroxene (3–5%); and opaque minerals (7–8%) with the size in the range of 0.1–4 mm with groundmass that is consisted of plagioclase microlite (2–3%); pyroxene (1%); opaque minerals (1%); and glass (40–56%).

**Fig. 3:**
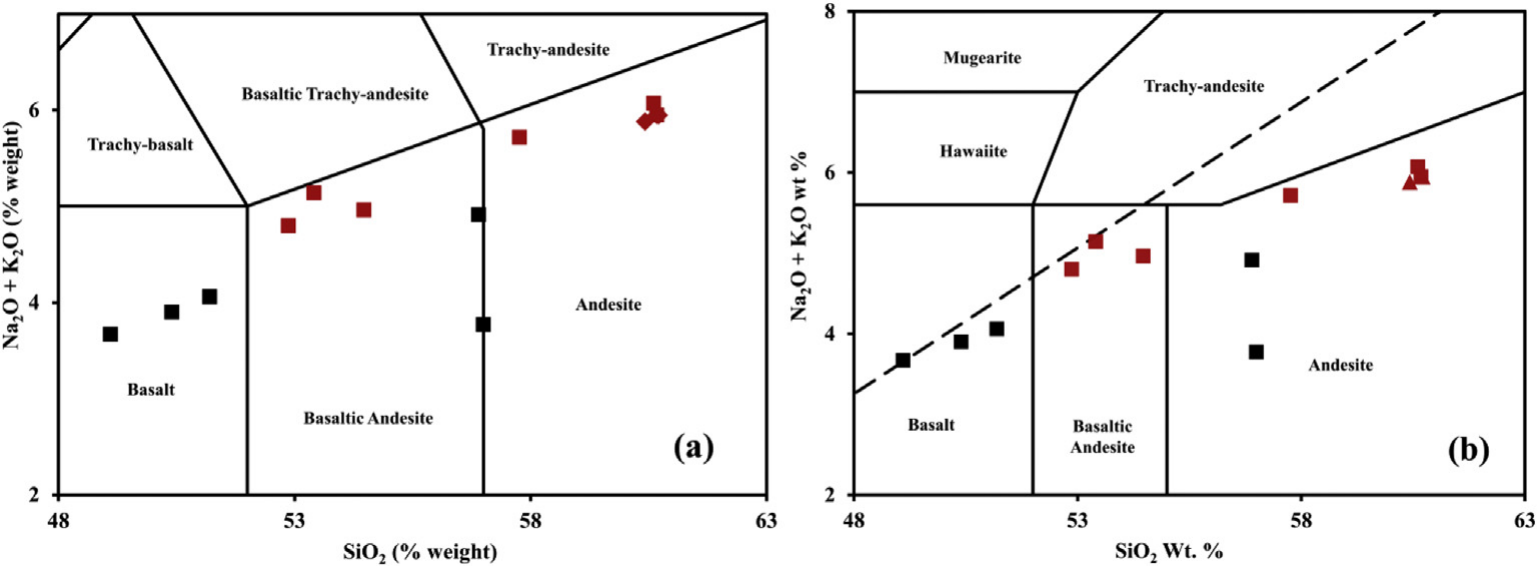
Plots of Na_2_O and K_2_O in respect with SiO_2_ in TAS diagram from (a) [5] and (b) [6]. Red squares indicate lava samples from the Ijen crater. Ijen crater samples from Ref. [1] are shown with black squares and Mount Blau samples are presented the red diamonds.

Table 2 shows the geochemistry data of the lava samples from the Ijen Crater and Mount Blau. All of the samples have Lol in the range of 0.13–1.94 %weight. This means that all of the samples are in fresh condition. Based on the total alkali silica (TAS) diagram (Fig. 3), lava samples from the Ijen Crater could be classified into two lithologies: basaltic andesite and andesite, meanwhile, lava samples from Mount Blau had andesite lithology. As a comparison, we also plotted the lava samples data from the Ijen Crater that were used in the previous study [1]. Major elements contents of the Ijen Crater lava samples from Ref. [1] are shown in the Table 3. Lava samples from the Ijen Crater obtained from Ref. [1] had basalt and basaltic andesite lithologies.

**Table 2:** Representative major and trace elements contents of the Mount Blau and Ijen Crater lava samples. StdErr shows the error values for each element.

Sample	BL A	BL B	BL C	IL3 B	IL3 E	IL3 F	IL3 H	IL3 J	IL3 K	StdErr
Major elements in %weight										
SiO_2_	60.68	60.42	60.71	60.60	52.87	53.41	57.76	54.47	60.68	0.250
TiO_2_	0.49	0.50	0.49	0.49	0.85	0.83	0.79	0.75	0.49	0.032
Al_2_O_3_	15.82	15.91	16.00	16.09	21.87	21.29	18.50	20.43	15.82	0.200
Fe_2_O_3_	7.06	7.07	6.81	6.77	9.22	9.14	8.58	9.34	7.06	0.120
MnO	0.13	0.14	0.10	0.14	0.17	0.17	0.16	0.18	0.13	0.007
MgO	2.79	2.70	2.79	1.93	1.22	1.44	1.61	1.66	2.79	0.070
CaO	6.87	7.16	6.94	7.64	8.77	8.34	6.71	7.92	6.87	0.120
Na_2_O	3.28	3.13	3.34	3.17	3.63	3.92	3.50	3.63	3.28	0.100
K_2_O	2.67	2.75	2.60	2.90	1.16	1.22	2.22	1.33	2.67	0.080
P_2_O_5_	0.22	0.22	0.21	0.26	0.22	0.24	0.17	0.29	0.22	0.011
LoI	0.46	0.83	0.52	0.67	0.70	1.94	0.50	0.13	0.46	
Total	100.46	100.83	100.52	100.67	100.7	101.94	100.5	100.13	100.46	
Trace elements in ppm										
Ba	0.0501	0.0528	0.0453	0.0619	0.0366	0.0281	0.043	0.0345	0.0371	0.0050
Cu	0.0474	0.0268	0.0032	0.0043	0.0102	0.0356	0.0052	0.0514	0.0057	0.0011
Zn	0.0054	0.0062	0.0051	0.0059	0.0069	0.0073	0.0062	0.0065	0.0064	0.0005
Rb	0.0059	0.0059	0.0057	0.0068	0.0026	0.0022	0.0058	0.0027	0.0031	0.0003
Sr	0.0260	0.0293	0.0280	0.0337	0.0467	0.0408	0.0329	0.0492	0.0510	0.0019
Co	0.0079	0.0125	0.0082	0.0051	0.0076	0.0058	0.1170	0.0077	0.0063	0.0006
Y	0.0023	0.0024	0.0021	0.0028	0.0020	0.0020	0.0024	0.0020	0.0023	0.0004
V	0.0148	0.0148	0.0151	0.0136	0.0190	0.0180	0.0176	0.0167	0.0138	0.0008

**Table 3:** Representative major elements contents of the Mount Blau and Ijen Crater lava samples based on the references [1,3].

Sample	IJ 1	IJ 2	IJ 3	IJ 4	IJ 5	KI 194	KI 162	KI 190	KI 29B	KI 75	KI 31D	KI 29D	KI 28
SiO_2_	51.20	57.00	49.10	56.90	50.40	57.21	58.30	50.81	50.47	51.65	49.76	58.53	57.88
TiO_2_	1.12	0.68	0.99	0.90	1.03	0.80	0.74	0.98	0.92	0.88	1.04	0.75	0.77
Al_2_O_3_	22.40	20.30	21.90	19.60	21.90	17.16	17.17	19.81	20.77	19.47	20.20	17.19	16.97
Fe_2_O_3_	9.81	7.80	10.5	7.85	10.10	7.66	7.41	10.23	9.71	9.71	9.45	7.13	7.41
MnO	0.15	0.10	0.13	0.12	0.13	0.15	0.16	0.18	0.18	0.20	0.16	0.16	0.15
MgO	2.54	2.21	2.23	2.56	1.84	2.93	2.72	3.95	3.56	3.47	3.64	2.07	2.82
CaO	8.22	6.07	8.22	6.30	8.30	6.70	6.32	9.91	10.28	9.05	9.39	8.13	6.41
Na_2_O	3.10	2.10	2.65	3.15	2.85	4.00	3.35	3.09	3.13	3.03	3.11	3.52	3.60
K_2_O	0.96	1.67	1.02	1.76	1.05	2.60	2.51	1.13	1.01	1.45	1.29	1.28	2.59
P_2_O_5_	0.25	0.19	0.23	0.19	0.22	0.22	0.22	0.23	0.24	0.24	0.28	0.29	0.19

Notes: IJ1; IJ2; IJ3; IJ4; IJ5 are Ijen Crater lava samples from Ref. [1]. KI 194; KI 162; KI 190; KI 29B; KI 75 are Ijen Crater lava samples from Ref. [3]. KI 31D; KI 29D; KI 28 are Mount Blau lava samples from Ref. [3].

The differences of geochemical characteristics of the samples used in Ref. [1] with the samples from this study could be influenced by the sampling locations. In the previous study [1], the samples which used only from one lava flow (Fig. 1), meanwhile, in this study we used samples from three lava flows. This data is also apt with the data in the other studies [[1], [2], [3]], which showed that lava samples from the Ijen Crater could be classified into three lithologies: basalt, basaltic andesite, and andesite. The data explained before also shows that the geochemical characteristics of the samples from the Ijen Crater are varied, whereas the samples from Mount Blau are relatively homogenous.

**Fig. 4:**
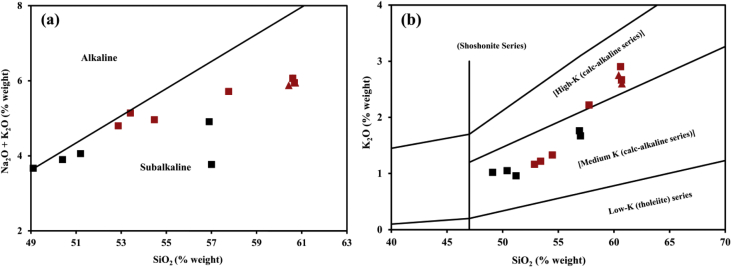
Magma series identification using (a) Plot of Na_2_O and K_2_O contents against SiO_2_ content in a TAS diagram from Ref. [5] and (b) Plot of K_2_O contents against SiO_2_ content in a magma series identification scheme from Ref. [7].

The magma characteristic from the Ijen Crater and Mount Blau are affiliated with moderate-K calc-alkaline to high-K calc-alkaline magma series (Fig. 4). Moreover, lava samples from both sources contain TiO_2_ less than 1.4 %weight, this indicates volcanic rocks that are derived from a subduction system [4]. The previous studies [1,3] obtained the same data.

**Fig. 5:**
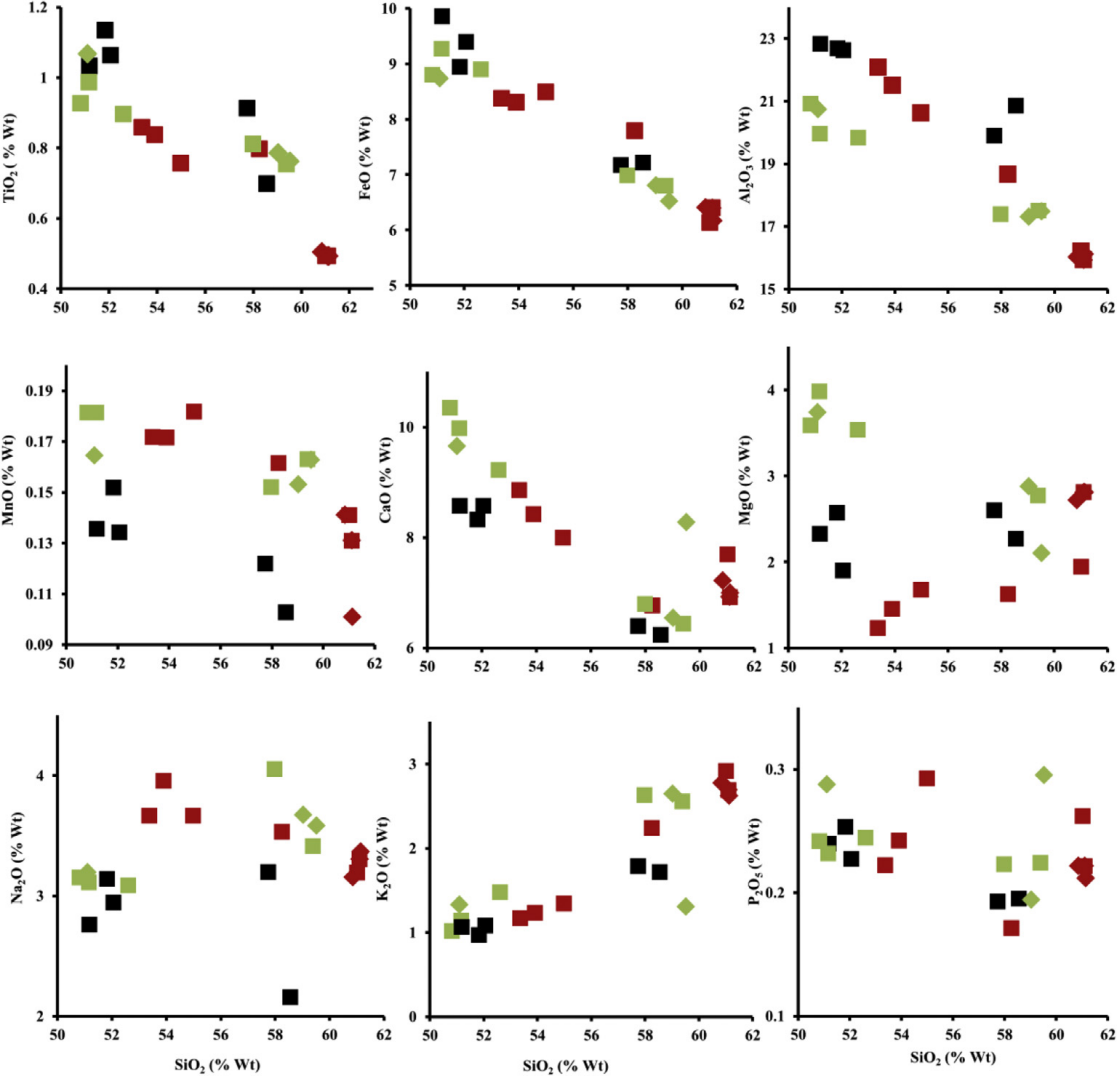
Plots of SiO_2_ content against different major elements oxide (Harker diagram) of the samples from the Ijen Crater (red squares), the Ijen Crater from previous study [1] (black squares) and [3] (green squares), Mount Blau (red diamonds), and the Mount Blau from previous study [3] (green diamonds).

Fig. 5 shows plot between some major element oxides and SiO_2_ content of all samples. As a comparison we also plotted the geochemical data of Ijen Crater and Mount Blau lava samples from previous study [1,3] showed in the Table 3. Fig. 5. Indicates that the major element oxides of the samples from Mount Blau are relatively similar. Meanwhile, negative correlations are observed between SiO_2_ and each of FeO, CaO, and Al_2_O_3_. In contrast, positive correlations are present between SiO_2_ and each of MgO and K_2_O (Fig. 5). These data relatively similar with the data from previous studies [1,3], except the correlation between MgO and Na_2_O with SiO_2_. In the [3], the correlation between MgO and SiO_2_ is negative, whereas between Na_2_O and SiO_2_ is positive. Meanwhile, in the present and previous data [1], the correlations between them are vice versa. The data indicate that MgO and Na_2_O content which were obtained in this research have a problem due to the contamination both in sample preparation process and XRF measurement. We suggest the readers to be cautious when using the data. But, the all data (except MgO and Na_2_O) are still in the good condition. These data broadly overlap with those of [3], and the error on MgO and Na_2_O doesn't seem to affect the other elements.

The percentage reduction of FeO along with the increasing percentage of SiO_2_ is caused by the formations of olivine and pyroxene minerals that contain a lot of Fe at the start of magma cooling. This is what caused magma differentiation to happen. The residual magma contains a higher percentage of SiO_2,_ while Fe content decreasing_._ On the other hand, the decreasing of CaO and Al_2_O_3_ can be the effect of the continuing formation of the plagioclase mineral from the start until the end of the magma cooling process [8,9]. The variation of the major element oxides diagrams in Fig. 5 shows that the magmatism process of the Ijen Crater is also controlled by magma mixing or contamination, based on the data obtained from Refs. [[1], [2], [3]].

## 2 Experimental design, materials, and methods

Sampling locations were chosen based on the geological map of the Ijen Volcanic complex [2] and followed the previous studies [1,10,11]. Samples were taken from three different lava flows that are part of lava flow 3 of the Ijen Crater (Il3) and one flow from the lava flow 3 of the Mount Blau (Bl3) [2]. Samples were taken from the unused outcrops from the previous studies [1,10,11]. These outcrops were chosen with the intention of complementing the data from the earlier studies.

A total of 20–50 g in each sample was crushed to the size of ≤50 μm using Jaw Crusher (Retsch, Germany) and Pulveriser (Fritsch, Germany). Geochemistry measurements were performed to get Lol data, major elements, and trace elements. The grain size should have a relatively similar size. The Lol measurements were done by heating the samples using muffle furnace with a temperature from 100 °C to 1000 °C. The samples were heated at 100 °C overnight, at 200 °C for 15 minutes, at 400 °C for 15 minutes, at 600 °C for 15 minutes, at 800 °C for 15 minutes, at 1000 °C for 20 minutes. Before the heating, samples' masses were measured using balance weighing with the accuracy ±0.0002 g and then stored in ceramic crucibles. After the heating at 1000 °C, the samples were left to cool down and then the masses were measured again to find the weight loss to calculate the Lol. Meanwhile, major elements and trace elements were measured using XRF (X-Ray fluorescence) method with the Thermo Scientific ARL 990 (Thermo Electron Corporation, Switzerland) in the Geological Survey Center Laboratory, Bandung, Indonesia. The error on major and trace elements are showed in the Table 2. All samples were also prepared in the form of thin sections with a thickness of 0.003 mm. These thin sections were used for mineralogy and rock texture analysis using Ci-POL polarizing microscope (Nikon, Tokyo, Japan) at the Petrographic Laboratory, Faculty of Earth Sciences and Technology, Institut Teknologi Bandung.
